# TAT-Protein Blockade during Ischemia/Reperfusion Reveals Critical Role for p85 PI3K-PTEN Interaction in Cardiomyocyte Injury

**DOI:** 10.1371/journal.pone.0095622

**Published:** 2014-04-21

**Authors:** Xiangdong Zhu, Zuo-Hui Shao, Changqing Li, Jing Li, Qiang Zhong, Jonathan Learoyd, Angelo Meliton, Lucille Meliton, Alan R. Leff, Terry L. Vanden Hoek

**Affiliations:** 1 Program in Advanced Resuscitation Medicine, Center for Cardiovascular Research, and Department of Emergency Medicine, University of Illinois Hospital and Health Sciences System, Chicago, Illinois, United States of America; 2 Section of Pulmonary and Critical Care Medicine, Department of Medicine, University of Chicago, Chicago, Illinois, United States of America; Thomas Jefferson University, United States of America

## Abstract

Recent work shows that cooling protection after mouse cardiac arrest and cardiomyocyte ischemia is mediated by Akt activation. The PI3K p85 subunit can either augment or inhibit Akt activation depending on its binding to p110 or PTEN respectively. To further clarify the role of PI3K p85 in cardioprotection, we studied novel TAT-p85 fusion proteins that selectively inhibit PI3K p85 binding. We hypothesized that TAT fused p85 lacking the PTEN binding site (TAT-ΔPTEN p85) would enhance Akt phosphorylation to afford cardioprotection. Conversely, TAT fused p85 lacking the p110 binding site (TAT-Δp110p85) would decrease Akt phosphorylation and abrogate cardioprotection. Microscopy and Western blot analysis demonstrated that TAT fusion protein was transduced into cardiomyocytes within 5 min and remained more than 2 h. Inhibition of PI3K/Akt by TAT-Δp110 p85 significantly increased cell death from 44.6±2.7% to 92.5±3.4% after simulated ischemia and reperfusion. By contrast, PTEN inhibition using TAT-ΔPTEN p85 decreased cell death to 11.9±5.3%, a similar level of cardioprotection seen with past cooling studies. Additional studies with the small molecule PTEN inhibitor VO-OHpic confirmed that PTEN inhibition was highly protective against cell death induced by ischemia and reperfusion. We conclude that blockade of p85-PTEN interaction and PTEN inhibition may be promising strategies for rescuing the heart from ischemia and reperfusion injury.

## Introduction

Sudden cardiac arrest (SCA) is a leading cause of death that is related to both global and focal ischemia/reperfusion (I/R) injury of the heart. While there are currently no drugs available that improve SCA survival, therapeutic hypothermia is a strategy that appears to improve heart function and survival after cardiac arrest. Our work and others suggest that therapeutic hypothermia protection is significantly mediated by enhanced phosphatidylinositol 3-kinase (PI3K) -Akt activation [1], [2], [3]. Given this role of PI3K-Akt in mediating one of the few life-saving treatments for SCA, new therapeutic approaches that optimize this survival response pathway during resuscitation of the ischemic heart could have enormous public health benefit.

Altered regulation of the PI3K signaling pathway contributes to many human diseases including cancer, diabetes, obesity, autoimmunity as well as I/R. A critical aspect of PI3K regulation that has recently emerged is the paradoxical ability of its p85α regulatory protein to bind and directly regulate not only the p110 catalytic subunit required for the stabilization and localization of p110-PI3K activity, but also the PTEN phosphatase that works simultaneously to deactivate the PI3K/Akt pathway [4], [5]. While PI3K-Akt is known to play a key role in cardioprotection against I/R injury [6], [7], a possible dual role of p85 (based upon its binding to p110 versus PTEN) in determining an optimal survival response of Akt activation during reperfusion has not been studied. Insights into a dual role for p85 during I/R could help guide new approaches for the resuscitation of ischemic heart tissue after both focal I/R and the global ischemic injury of SCA.

For this study we constructed a TAT fusion protein, TAT-ΔPTEN p85, that contains the TAT protein transduction domain fused to a p85 subunit of PI3K lacking a PTEN binding site. The N-terminal SH3-BH region of p85α (amino acids 1–313) has been identified as the region that mediates PTEN binding and regulation. Cellular expression of the ΔPTEN binding site of p85 results in a substantially increased magnitude and duration of p-Akt levels in response to growth factor stimulation [4]. We used an established cardiomyocyte I/R model that has previously demonstrated Akt-mediated hypothermia protection to test whether TAT fusion protein alteration of p85 binding can affect similar levels of cardioprotection. We hypothesized that TAT-ΔPTEN p85 would interfere the endogenous p85 binding to PTEN, resulting in reduced PTEN activity as evidenced by enhanced p-Akt phosphorylation. To test the dual role of p85 binding during I/R, we also used the TAT fusion protein TAT-Δp110p85 (known as dominant negative p85 or Δp85 previously) that inhibits endogenous p85 binding to p110 catalytic subunits and has been shown to decrease tissue p-Akt [8], [9].

To further confirm the role of PTEN inhibition in cardioprotection, we investigated whether the small molecule PTEN inhibitor VO-OHpic [10] similarly protects cardiomyocytes against I/R injury. Collectively our results show that p85 binding to p110 versus PTEN may critically determine the phenotype of cardiomyocyte I/R injury. Strategies that alter p85 binding during I/R to optimize PTEN inhibition and Akt activation may achieve levels of cardioprotection seen in prior studies of therapeutic hypothermia.

## Materials and Methods

### Ethics Statement

The investigation conforms to the Guide for the Care and Use of Laboratory Animals, published by the National Institutes of Health (NIH Publication No. 85-23, Revised 1996). The procedure for cardiomyocyte isolation was approved by the University of Illinois at Chicago's Institutional Animal Care and Use Committee (Permit Number: 11-198).

### Materials

The PTEN inhibitor VO-OHpic was obtained from Sigma (St. Louis, MO). Propidium iodide (PI) for measuring cell death was obtained from Invitrogen (Carlsbad CA). The primary antibodies, including Akt, pAkt-S473, pAkt-T308 were purchased from Cell Signaling Technology (Beverly, MA). The secondary antibodies include horseradish peroxide conjugated anti-mouse and anti-rabbit antibodies from Amersham (Arlington Heights, IL). Polyclonal anti-p85 subunit of PI3K was purchased from Upstate Biotechnology (Lake Placid, NY). TAT-Δp110 p85, a dominant-negative form of the class IA PI3K adaptor subunit with the deletion of 35 amino acids (residues 479–513, formerly named as Δp85) of its p110 binding site, was produced in our laboratory as described previously [8], [9], [11], [12]. BL21 *Escherichia coli* was obtained from Novagen (Madison, WI). pGEX vectors containing wild type p85 and Δp110-p85 cDNA were gifts from Dr. M. Kasuga (Kobe University Graduate School of Medicine, Kobe, Japan). pTAT and pTAT-green fluorescent protein (GFP) were gifts from Dr. S. Dowdy (University of California San Diego, La Jolla, CA).

### Generation of TAT-ΔPTEN p85 Fusion Protein

To construct the pTAT-ΔPTEN p85 plasmid, a cDNA fragment encoding ΔPTEN p85 (deletion of amino acids 1–313 from p85) was amplified by PCR from the wild type p85 cDNA in pGEX with the following primers (sense, 5′- CAC ACC GGT CCC ACT ACT GTA GCC AAC AAC GGT ATG –3′; antisense, 5′- CAC GAA TTC TCA TCG CCT CTG CTG CGC GTA CAC -3′). The PCR products have *Age*I and *Eco*RI cutting sites plus 3 extra bases for efficient cutting before and after ΔPTEN p85 cDNA, respectively. PCR was carried out for 35 cycles using 0.5 min of denaturation at 95°C, 0.5 min of annealing at 62°C, and 1.5 min of extension at 72°C. The amplified PCR products were digested with *Age*I/*EcoR*I, purified and ligated into an *Age*I/*EcoR*I digested pTAT vector using T4 ligase. The final sequence was verified by an Automatic Sequencer.

Purification of TAT fusion proteins was performed as previously described [13]. BL21(DE3)pLysS-competent *Escherichia coli* were transformed with either pTAT-ΔPTEN p85 or pTAT-GFP constructs, and TAT fusion protein was induced with 1 mM isopropyl β-D-1-thiogalactopyranoside overnight at room temperature and was purified using a native isolation method. Briefly, *E. coli* cells were sonicated in Tris-buffered saline (TBS) plus protease inhibitor cocktail (Roche, Indianapolis, IN) and centrifuged, and the resulting supernatant was loaded onto a Ni-ProBond resin column (Invitrogen, Carlsbad, CA). The column was washed with 100 mL TBS containing 75 mM imidazole, and 1 L of TBS containing 0.1% Triton X-114 to remove endotoxin [14], and 1L of TBS without detergent at 4°C. Pure TAT fusion proteins with N-terminal hexa-His tags were eluted with an imidazole gradient, desalted on a PD-10 column into TBS, and stored at −80°C before use.

### Primary culture of mouse cardiomyocytes

Primary cultures of mouse ventricular cardiomyocytes were prepared from hearts of 1 to 2-day-old neonatal C57BL6/J mice (Jackson, Bar Harbor, ME) as described previously [1]. In brief, the cells were isolated at 37°C for 8 min with 0.1% trypsin in Hanks' balanced salt solution (HBSS) without Ca^2+^ and Mg^2+^ (pH 7.4). The first cell suspension was discarded, whereas the subsequent suspensions were added to trypsin inhibitor solution in cold HBSS with Ca^2+^ and Mg^2+^ (pH 7.4) until all cardiac cells were isolated (5 to 6 cycles). To remove fibroblasts, the isolated cells were preplated for 90 min at 37°C. The resulting supernatants were then centrifuged and plated at a density of 0.6×10^6^ on laminin-coated coverslips with MEM supplemented with 10% fetal bovine serum (Invitrogen, Carlsbad, CA), 50 U/ml penicillin, and 1.5 µM vitamin B_12_ (Sigma, St. Louis, MO). Myocyte purity was ∼90% as determined by immunofluorescent staining for α-sarcomeric actin and myosin heavy chain (Sigma, St. Louis, MO). Experiments were performed on 6–8 day cultures.

### Simulated ischemia and reperfusion

Synchronously contracting cardiomyocytes on glass coverslips were equilibrated for 30 min in a Balanced Salt Solution (BSS) containing 117 mM NaCl, 4 mM KCl, 18 mM NaHCO_3_, 0.76 mM MgSO_4_, 1.0 mM NaH_2_PO_4_, 1.21 mM CaCl_2_ and 5.6 mM Glucose, then subjected to simulated ischemia for 90 min in a ischemia solution that was placed in a hypoxic chamber (0.5% O_2_, 20% CO_2_ and 79.5% N_2_) followed by 3 h reperfusion at 37°C. Ischemia for 90 min and reperfusion for 3 h was selected for these studies because it was the optimum time to induce ∼50% death of cardiomyocytes [1]. Ischemic BSS contained no glucose plus 2-deoxyglucose (20 mM) to inhibit glycolysis, 8.0 meq/l [K^+^], and bubbled with 80% N_2_ - 20% CO_2_ for 45 min to produce BSS with 3–5 Torr PO_2_, 144 Torr PCO_2_, and pH 6.8. After the ischemic challenge, cells were incubated with normoxic BSS (21% O_2_, 5% CO_2_, and 74% N_2_) to simulate reperfusion [15].

### Cell death measurement

Cell death was assessed under a fluorescent microscope (Nikon Ti-E, Nikon Instruments) with the exclusion dye propidium iodide (5 µM; Sigma, St Louis, MO) as measured at 540 nm excitation and 590 nm emission [1]. At the end of 3 h reperfusion, the cells were permeabilized with digitonin (300 µM, Sigma, St Louis, MO). Cell death was expressed as the propidium iodide fluorescence relative to the maximal value seen after digitonin exposure at the end of reperfusion (100%).

### Western blot analysis

The cells were lysed in buffer containing 1% Triton X-100, 20 mM Tris, 137 mM NaCl, 2 mM EDTA, 10% glycerol, 10 mM sodium pyrophosphate, 50 mM NaF, 1 mM Na_3_VO_4_, 200 mM PMSF, and 1× protease inhibitor cocktail. Protein quantification was performed with a Bradford assay (Bio-Rad, Hercules, CA). Protein lysate (30 µg/lane) was loaded and resolved on a 10% SDS-PAGE gel and was then transferred to a nitrocellulose membrane. After the membranes were blocked in 5% fat-free milk in TBS-Tween 20, they were probed with antibodies against phosphorylated Akt (Thr308 and Ser473) and total Akt. Signals were amplified and visualized with horseradish peroxidase-conjugated secondary antibody and enhanced chemiluminescence. Band quantification was performed using Image J software (National Institutes of Health, USA) and normalized to loading controls.

### Fluorescence microscopy

Cardiomyocytes were incubated with 1 µM FITC-conjugated TAT for up to 120 min at 37°C, and then were washed three times with BSS buffer. Cells were fixed in 2% paraformaldehyde in phosphate-buffered saline (PBS) for 30 min, washed three times in PBS. Cells were examined under a fluorescent microscope (Olympus AX70 Olympus UK Ltd, Watford, UK) with ×400 magnification using Smart capture VP imaging software (Digital Sciences Ltd, Cambridge, UK).

### Statistical analysis

Results were expressed as means ± SEM. A field of ∼500 cells was observed in each experiment for cell death measurement. For comparison among the different treatment groups, one-way ANOVA were used with post hoc examination by Fisher's least significant difference test. *P*<0.05 was considered statistically significant.

## Results

### Efficacy of TAT-Δp110 p85 in blocking PI3K-Akt activation

**Figure 1:**
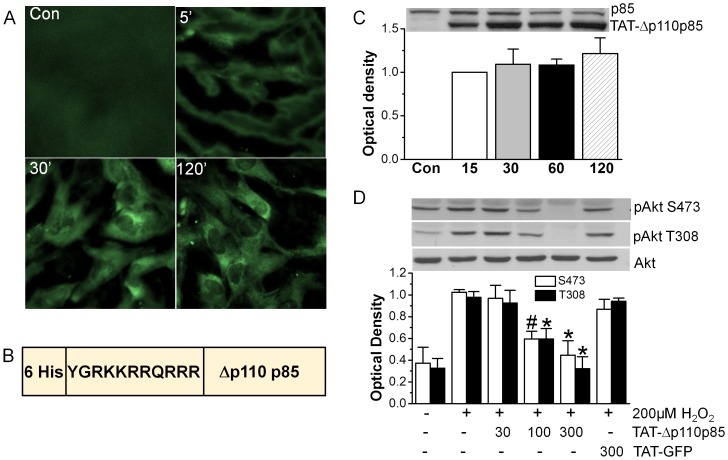
Inhibition of H_2_O_2_- induced Akt activation by TAT-Δp110 p85. A. TAT protein transduction in cardiomyocytes as detected by fluorescent microscopy. Cardiomyocytes were incubated with FITC-conjugated TAT for 0–120 min, fixed and observed under microscopy. A representative image is shown (n = 3). B. The schematic structure of TAT-Δp110 p85 fusion protein, consisting of 6 histidines, 11 amino acids of TAT sequence, and the p85 mutant lacking p110 binding site. C. Time-course of TAT protein transduction detected by Western blot. Cardiomyocytes were incubated with 300 nM TAT-Δp110 p85 for ≤120 min, and its expression in cardiomyocytes was detected by anti-p85 antibody. These results are representative of four different experiments. D. Cardiomyocytes were incubated with 30–300 nM TAT-Δp110 p85 for 20 min before stimulation with 200 µM H_2_O_2_ for 20 min. Akt phosphorylation and Akt expression was detected by specific antibodies (n = 3).

To assess the efficacy of TAT protein transduction in cardiomyocytes, FITC-conjugated TAT was used to determine the localization of TAT protein under fluorescence microscope. At 5 min, TAT protein was mostly associated with the plasma membrane (Fig. 1A). By 30 min TAT protein was visualized throughout heart cells, and the majority of TAT protein localized to the cytoplasm. Cytosolic staining of TAT protein was still observed after 120 min from initial treatment with TAT protein.

We then determined whether TAT-Δp110 p85 was able to inhibit Akt activation in cardiomyocytes. Fig. 1B showed the structure of TAT-Δp110 p85, which consist of 6 histidines for protein purification, 11 amino acids of TAT sequence for protein transduction, and the p85 mutant lacking p110 binding site. Western blot analysis was used to determine the time course of TAT-Δp110 p85 transduction in cardiomyocytes. Consistent with results from microscopic study, transduction of TAT-Δp110 p85 was observed at 15 min, and intact protein was still present at 120 min (Fig. 1C). TAT-Δp110 p85 had no effect on Akt phosphorylation in unstimulated states (data not shown). However, it inhibited Akt phosphorylation caused by 200 µM H_2_O_2_ in a concentration-dependent fashion. Akt phosphorylation was reduced at 100 nM and blocked completely with 300 nM TAT-Δp110 p85 (Fig. 1D). By contrast, 300 nM TAT-GFP control had no effect on Akt phosphorylation caused by H_2_O_2_. Therefore, 300 nM of TAT-Δp110 p85 was used in the subsequent experiments. These results demonstrated that TAT-Δp110 p85 inhibited Akt phosphorylation after transduction into cardiomyocytes.

### Effect of TAT-Δp110 p85 on cell death after simulated I/R

**Figure 2:**
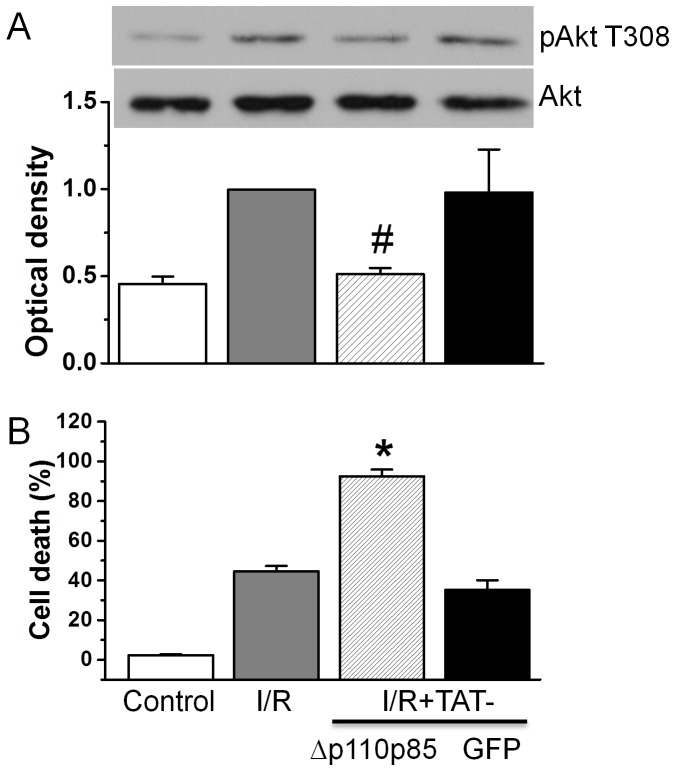
Effect of TAT-Δp110 p85 on Akt phosphorylation and cardiomyocyte injury caused by I/R. Cardiomyocytes were added with 300-Δp110 p85 and then undergone simulated I/R. Akt phosphorylation and expression was measured by Western blot (A) and cardiomyocyte injury was measured by propidium iodide staining (B). The values presented were the mean ± SEM from 3 independent experiments. #p<0.05 and *p<0.01 vs. I/R alone.

We then examined whether TAT-Δp110 p85 attenuated phosphorylation of Akt under I/R conditions. Compared to BSS equilibration buffer control, I/R (ischemia for 90 min followed by 30 min reperfusion) significantly increased Akt phosphorylation (Fig. 2A). Treatment with 300 nM TAT-Δp110 p85, but not TAT-GFP blocked Akt phosphorylation under I/R conditions.

We next examined the functional consequence of Akt inhibition by TAT-Δp110 p85 in cardiomyocytes exposed to I/R. Cardiomyocytes were pretreated with 300 nM TAT-Δp110 p85 or TAT-GFP before exposed to I/R. I/R induced significantly cell death from 2.3±0.4% of baseline control to 44.6±2.7%. Compared to I/R alone, TAT-Δp110 p85 increased cell death to 92.5±3.4% (*p*<0.01 vs. I/R alone, Fig. 2B). By contrast, TAT-GFP has no effect on cardiomyocyte injury compared to I/R alone. These results supported the notion that p85 binding to p110 during I/R is critical for heart cell survival during I/R.

### Effect of TAT-ΔPTEN p85 on Akt activation

**Figure 3:**
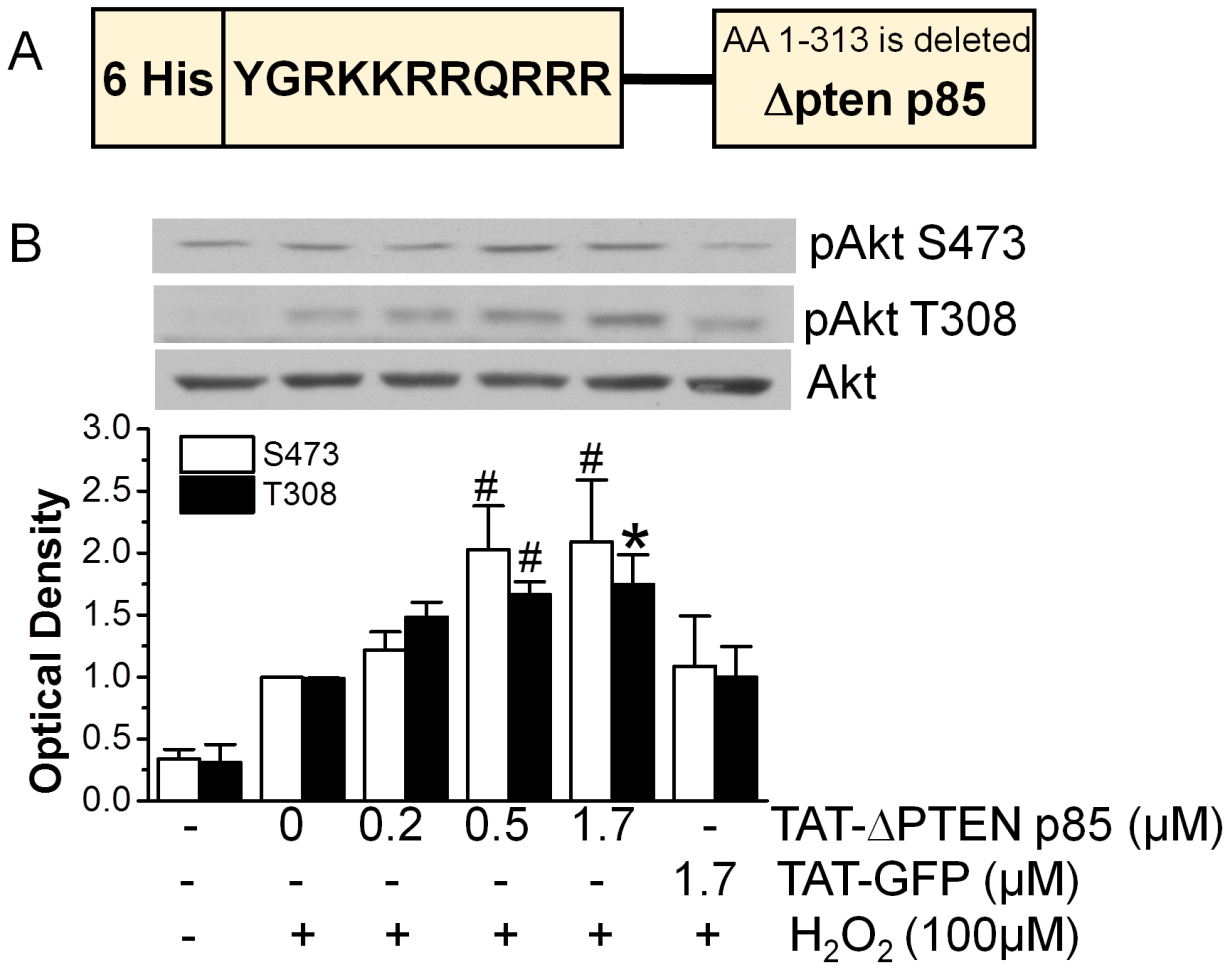
Enhancement of H_2_O_2_- induced Akt activation by TAT-ΔPTEN p85. A. The schematic structure of TAT-ΔPTEN p85 fusion protein, consisting of 6 histidines, 11 amino acids of TAT sequence, and the p85 mutant lacking PTEN binding site. B. Cardiomyocytes were incubated with indicated concentrations of TAT-ΔPTEN p85 for 20 min, and then stimulated with H_2_O_2_ for another 20 min. Phosphorylation of Akt in cardiomyocytes was detected by phosphorylation specific antibody. Equal loading of Akt was confirmed by stripping and reprobing with Akt antibody. These results are representative of four different experiments.

Recent work has shown that p85 not only binds to p110 PI3K, but also binds to PTEN and enhances PTEN lipid phosphatase activity [4]. Based on this information we have constructed and purified TAT-ΔPTEN p85 that lacks the N-terminal PTEN binding site. Fig. 3A showed the structure of TAT-ΔPTEN p85 consisting of 6 histidines for protein purification purpose, 11 amino acids of TAT sequence for protein transduction and the p85 mutant lacking the N-terminal PTEN binding site (amino acids 1–313 of SH3-BH domain).

Akt activation is a specific marker for PTEN inhibition and has been used to indicate the inhibitory potency of PTEN inhibitors [16], [17], as PTEN exhibits a high phosphatase specificity towards 3-phosphorylated phosphoinositides that activate Akt. As shown in Fig. 3B, TAT-ΔPTEN p85 enhanced Akt phosphorylation caused by H_2_O_2_ in a concentration-dependent fashion. For these studies a lower concentration of H_2_O_2_ (100 µM) was used in order to test for possible enhancement of Akt activation. Enhanced Akt phosphorylation was observed at 0.5 µM and further increased with 1.7 µM TAT-ΔPTEN p85. By contrast, 1.7 µM TAT-GFP had no effect on Akt phosphorylation caused by H_2_O_2_. These results demonstrated that TAT-ΔPTEN p85 enhanced Akt phosphorylation after transduction into cardiomyocytes.

### Effect of TAT-ΔPTEN p85 on the death of cardiomyocytes after simulated ischemia and reperfusion

**Figure 4:**
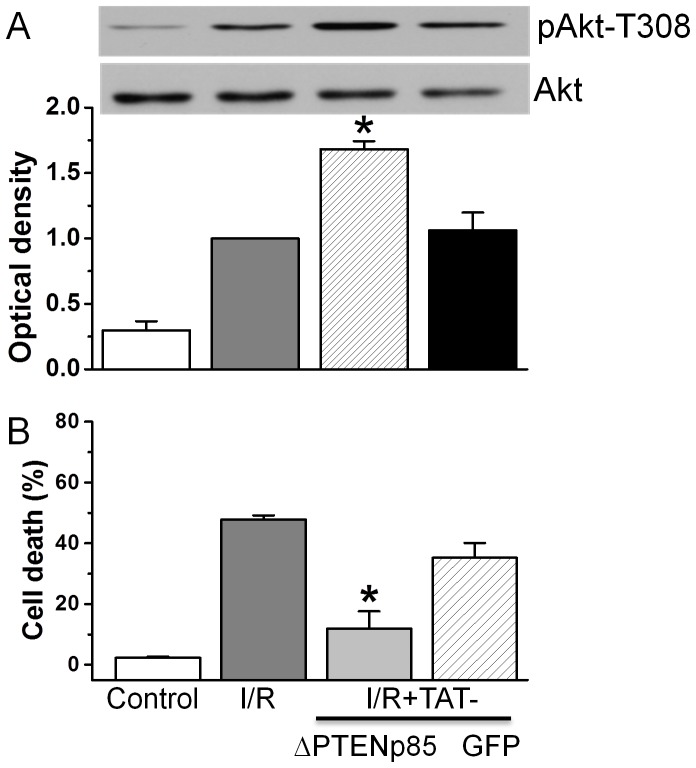
Effect of TAT-ΔPTEN p85 on Akt activation and cardiomyocyte injury caused by I/R. A. Cardiomyocytes were added with 1.7 µM TAT-ΔPTEN p85 or 1.7 µM TAT-GFP, prior to simulated ischemia for 90 min followed by 30 min reperfusion (I/R) at 37°C. Expression and phosphorylation of Akt in cardiomyocytes was detected by specific antibody. The values presented were the mean ± SEM from 3 independent experiments. B. Cardiomyocytes were added with 1.7 µM TAT-ΔPTEN p85 or 1.7 µM TAT-GFP, prior to simulated ischemia for 90 min followed by 3 h reperfusion (I/R) at 37°C. Cardiomyocyte injury was measured by propidium iodide staining. The values presented were the mean ± SEM from 3 independent experiments. *p<0.01 vs. I/R alone.

We next examined whether TAT-ΔPTEN p85 enhanced phosphorylation of Akt under I/R conditions. Compared to BSS equilibration buffer control, I/R (ischemia for 90 min followed by 30 min reperfusion) significantly increased Akt phosphorylation (Fig. 4A). TAT-ΔPTEN p85, but not TAT-GFP further increased Akt phosphorylation under I/R conditions.

We then examined the functional consequence of Akt activation by TAT-ΔPTEN p85 on cardiomyocyte survival after exposed to I/R. Cardiomyocytes were pretreated with 1.7 µM TAT-ΔPTEN p85 or TAT-GFP immediately before exposed to I/R. I/R caused cell death from 2.3±0.4% of baseline control to 47.8±1.4%. Compared to I/R alone, TAT-ΔPTEN p85 attenuated cell death to 11.9±5.3% (Fig. 4B, *p*<0.01). TAT-GFP has no effect on cardiomyocyte death compared to I/R alone. These results supported a dual role for p85 binding during cardiomyocyte I/R, with decreased p110 binding associated with cell death and decreased PTEN binding (i.e. PTEN inhibition) associated with improved heart cell survival.

### Effect of a chemical inhibitor of PTEN, VO-OHpic, on Akt activation and cardiomyocyte injury after I/R

**Figure 5:**
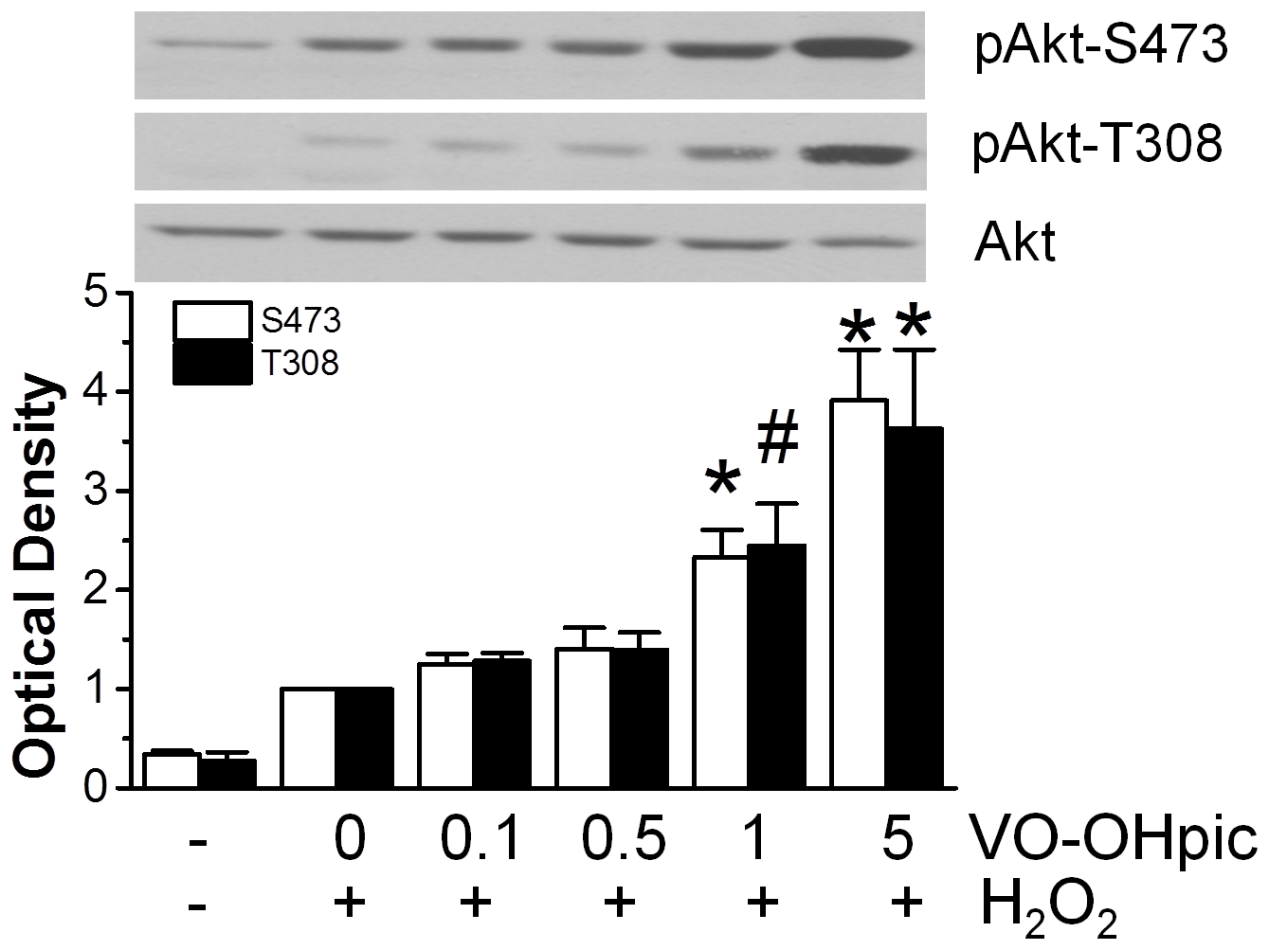
Enhancement of H_2_O_2_- induced Akt activation by PTEN inhibitor VO-OHpic. Cardiomyocytes were incubated with indicated concentrations of VO-OHpic for 20 min, and stimulated with or without 100 µM H_2_O_2_ for 20 min. Phosphorylation of Akt in cardiomyocytes was detected by phosphorylation specific antibody. Equal loading of Akt was confirmed by stripping and reprobing with Akt antibody (n = 4). #*p*<0.05 and *p<0.01 vs. H_2_O_2_ alone.

VO-OHpic is a vanadium inhibitor of PTEN with nanomolar affinity in vitro and in vivo [10], [18]. Among the reported PTEN inhibitors, VO-OHpic was the most potent and specific compound [10], which has subsequently been employed to probe the role of PTEN in PI3K-dependent signaling in the heart [16]. To confirm that PTEN inhibition is cardioprotective, we first determined the concentration-dependent effect of VO-OHpic on Akt phosphorylation in mouse cardiomyocytes stimulated with the submaximal concentration of H_2_O_2_. VO-OHpic at 1–5 µM augmented phosphorylation of Akt induced by H_2_O_2_ (Fig. 5).

**Figure 6:**
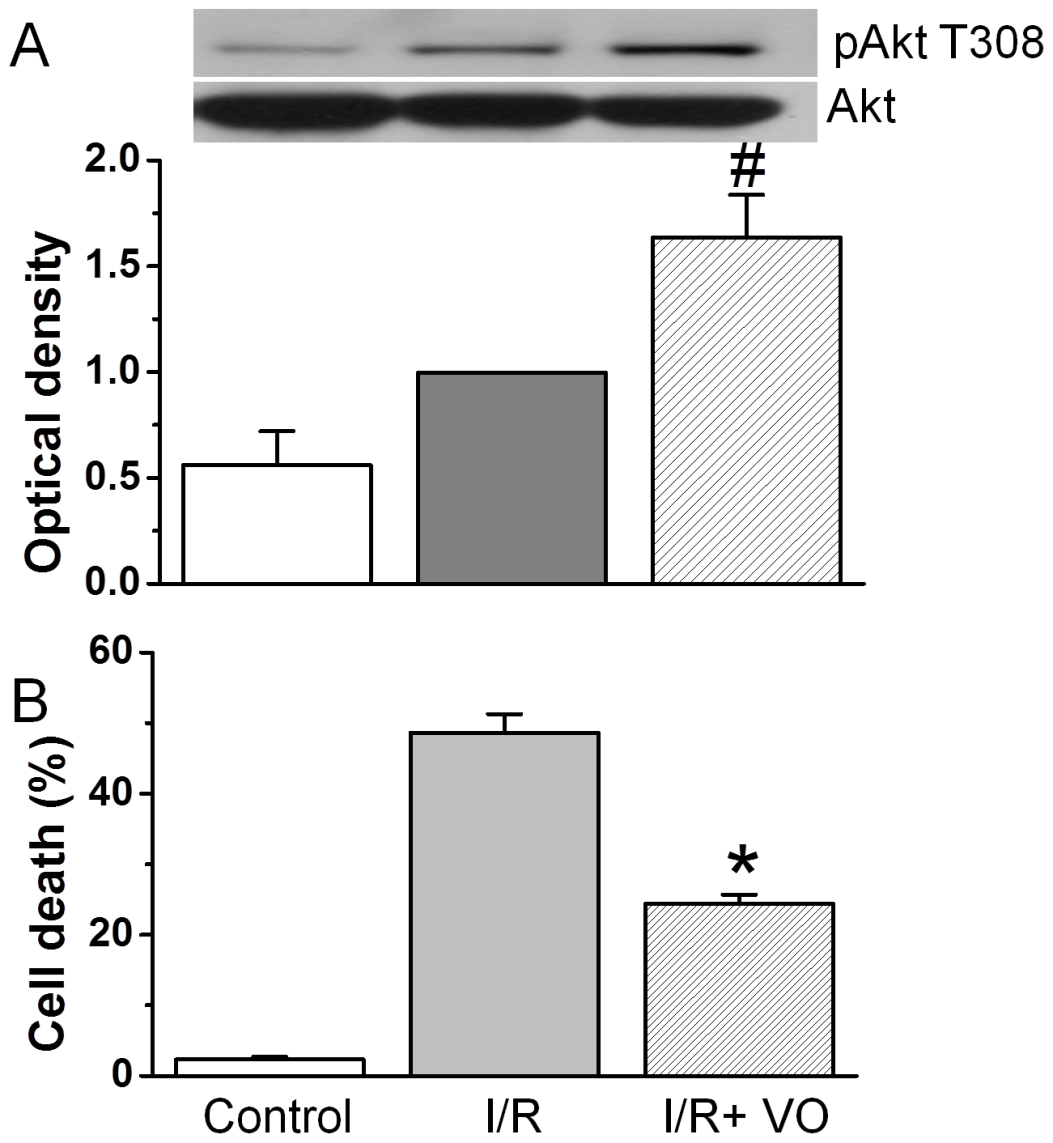
Effect of VO-OHpic on phosphorylation of Akt and cardiomyocyte injury caused by I/R. A. Cardiomyocytes were added with or without 1 µM VO-OHpic prior to simulated ischemia for 90 min followed by 30 min reperfusion at 37°C. Expression and phosphorylation of Akt in cardiomyocytes was detected by specific antibody. These results are representative of four different experiments. B. Cardiomyocytes were added with 1 µM VO-OHpic and were subjected to simulated ischemia for 90 min followed by 3 h reperfusion at 37°C. Cardiomyocyte injury was measured by propidium iodide staining. The values presented were the mean ± SEM from 3 independent experiments. #*p*<0.05 and *p<0.01 vs. I/R alone.

We then examined whether VO-OHpic enhanced phosphorylation of Akt under I/R conditions. Compared to non-ischemic equilibration buffer control, I/R significantly increased Akt phosphorylation (Fig. 6A). VO-OHpic at 1 µM further increased Akt phosphorylation under I/R conditions. We did not choose 5 µM because it has been reported to lose its specificity at higher concentrations [10], [18].

We next examined the functional consequence of Akt activation by VO-OHpic in cardiomyocytes. Cardiomyocytes were treated with 1 µM VO-OHpic before simulated I/R. I/R increased cell death from 2.3±0.4% of baseline control to 48.7±2.6%. VO-OHpic decreased I/R induced cell death to 24.4±1.3% (Fig. 6B, *p*<0.01).

## Discussion

**Figure 7:**
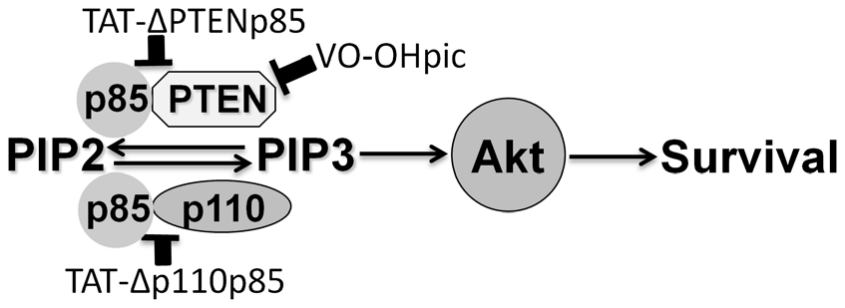
Schematic of p85 regulation of either catalytic subunit of PI3K or PTEN on Akt phosphorylation and cardiomyocyte survival after exposure to I/R. p85 binding to p110 increase Akt phosphorylation and results in decreased cell death after exposure to I/R, while p85 binding to PTEN decreases Akt phosphorylation and results in increased cell death. By modulating p85 binding to its cognitive binding partners, cell survival is modulated by TAT fusion proteins with p85 mutants lacking either p110 or PTEN binding site. PTEN inhibition by VO-OHpic has a similar cardioprotective effect as for TAT-ΔPTEN p85.

The present study demonstrated that 1) inhibition of p85 binding to PI3K catalytic subunit p110 by TAT-Δp110 p85 resulted in decreased Akt phosphorylation and increased cell death after I/R, 2) inhibition of p85 binding to PTEN by TAT-ΔPTEN p85 in cardiomyocytes increased Akt activation and attenuated I/R induced cell death, and 3) compared to TAT-ΔPTEN p85, chemical inhibition of PTEN by VO-OHpic has a similar protective effect on I/R injury. This data supports a dual role for p85 in regulating Akt activation during I/R via binding either to the PI3K catalytic subunit p110 versus the phosphatase PTEN. Blocking p85 binding to p110 results in decreased Akt phosphorylation and enhanced I/R injury of cardiomyocytes, while blocking its binding to PTEN increased Akt activation and decreased death of cardiomyocytes exposed to I/R (Fig. 7). In summary, these results suggests that PTEN inhibition during I/R, either by TAT-fusion protein inhibition of p85-PTEN binding or by direct PTEN inhibition may be promising approaches for cardioprotection during I/R.

### TAT-Δp110 p85 on Akt inactivation and cell death after I/R

Considerable research has been undertaken to investigate the possibility of increasing cell survival and reducing cell death following ischemia and reperfusion. Studies have shown the essential role of PI3K in activating Akt with downstream activation of pro-survival kinases and suppression of pro-apoptotic pathways [6]. Optimal activation of the PI3K/Akt survival signaling pathway at the time of reperfusion confers protection against reperfusion-induced injury [19]. This is consistent with our prior work which suggests that therapeutic hypothermia protection is associated with enhanced activation of this pathway within minutes of reperfusion [1]. However, without pharmacological manipulation, the Akt activation during reperfusion is not sufficient to protect the heart from I/R injury. PTEN activation due to p85 binding may be a critical reason why Akt activation during I/R is insufficient to optimally mediate survival. Consistent with this, in the current study we observed 40% cell death after reperfusion despite a significant increase in Akt phosphorylation at 30 min of reperfusion (Fig. 2A, second lane). The TAT-Δp110 p85 used in this current study has been used successfully to inhibit Akt activation in the lung [9], [12]. Similar to this past work, we found that TAT-Δp110 p85 reduced Akt activation in cardiomyocytes and increased cell death from 40% to 90% after exposure to I/R (Fig. 2). These results suggest that Akt activation during reperfusion seen in this study is critical for heart cell survival. While this is consistent with other work showing that pharmacologic inhibitors of PI3K such as wortmannin and Ly294002 increase cardiomyocyte injury caused by I/R [20], [21], there are limitations to establishing the role of PI3K during I/R given the non-specific effects of many of these inhibitors. Studies have shown that wortmannin also exhibits an inhibitory effect on cPLA_2_ [22], phospholipase D [23], or myosin light chain kinase [24]. This report is one of the first to use a TAT-fusion protein approach to specifically inhibit class 1A PI3K in an I/R model of heart cell injury. Furthermore, this study highlights a possible critical role of p85 during I/R due to its relative binding of p85 to PTEN and optimal Akt-mediated survival responses.

### TAT-ΔPTEN p85 on Akt and heart cell survival after I/R

As the key negative regulator of the PI3K/Akt pathway, PTEN is an important target for cardioprotection in I/R injury. Previous studies found that PTEN inactivation in neonatal cardiomyocytes by overexpression of dominant negative PTEN H123Y mutant activates the Akt prosurvival pathway, reduces apoptosis, and increases survival [25]. Similarly, it has also been shown that a cardiac specific knockout of PTEN reduced infarct size, blunted cardiomyocyte apoptosis and improved post I/R contractile function in isolated heart [26] and in animals [27] subjected to I/R injury. The diminished PTEN activity in conditional knockout mice lacking p85α has revealed a previously unknown p85α-dependent negative-feedback pathway that controls PI(3,4)P_2_ and PI(3,4,5)P_3_ level by regulating PTEN [28]. In line with these studies, we showed that TAT-ΔPTEN p85 fusion protein enhanced Akt activation in cardiomyocytes (Fig. 3) and attenuated cell death caused by I/R (Fig. 4). Our results further support the negative-feedback pathway of p85α in controlling Akt activation by regulating PTEN activity [5], [29].

### PI3K isoform activation during ischemic reperfusion

PI3K isoforms convey distinct roles in cardiac physiology and development. Class IA PI3K (p110α, β, δ) regulates cardiomyocyte growth and apoptosis, whereas class 1B PI3Kγ downregulates cardiac contractility through inhibiting cyclic adenosine monophosphate production [30], [31]. The specific PI3K isoform responsible for Akt activation and cardioprotection during I/R injury has not been clearly defined. Prior studies using pharmacological inhibitor wortmannin or Ly294002 have demonstrated the requirement of PI3K activity in protecting heart form reperfusion injury [7], [32]. As these drugs are pan- PI3K inhibitors, the specific isoform of PI3K involved in reperfusion injury could not be determined. As cardioprotection could be achieved through either growth factors (IGF-1, insulin, fibroblast growth factor-2) that activate class 1A PI3K or G-protein receptor ligands (such as bradykinin, adenosine agonists) that activate PI3Kγ [6], it is speculated that all of these PI3K isoforms are involved in cardioprotection from I/R injury. Our results showed that TAT-Δp110 p85 increased cardiomyocyte death from 40% to 90% after exposed to I/R (Fig. 2), supporting the critical role of class 1A PI3K in maintaining cardiomyocyte survival. Recent studies found that PI3Kγ knockout mice had reduced Akt phosphorylation and increased infarction size after reperfusion, suggesting that PI3Kγ may not only regulates contractility but also cell survival [33], [34]. Studies with isoform-selective PI3K inhibitors are needed to clarify the specific role of each PI3K isoform in cardioprotection during ischemic reperfusion. PI3K inhibitors targeting p110α (PIK75), β (TGX221), γ (AS252424) and δ (IC87114) has been successfully used in studies to identify the role of PI3K isoform (s) involved in osteoclast survival [35]. Furthermore, our results could also be supported by studies using transgenic mice overexpressing mutant p85α lacking either PTEN- or P110- binding site.

### VO-OHpic on Akt and cell survival after I/R

In this study, we also showed that pharmacological inhibition of PTEN with VO-OHpic ameliorated reperfusion injury and reproduced the cardioprotective effects obtained by TAT-ΔPTEN p85. The VO-OHpic action on PTEN is confirmed by the increased phosphorylation of Akt, a downstream target of PTEN-dependent signaling, caused by H_2_O_2_ or I/R (Figs. 5 and 6). This is in line with a growing body of evidence indicating that cardioprotection could be achieved by reducing the activity or expression of PTEN, the key negative regulator of the PI3K pathway [26], [36], [37], [38]. Akt upregulation by a first generation of PTEN inhibitor (bpV) has also been shown recently to prevent ischemic injury in brain [39], [40] and liver [41]. In addition, it has been reported that myocardial infarct size was significantly reduced in VO-OHpic-pretreated mice [16].

It is important to recognize some limitations of our findings. We used neonatal cardiomyocytes that may not entirely reflect the response of adult cardiomyocytes in the intact heart following I/R. Neonatal cardiomyocytes were chosen over adult cardiomyocytes in this study because of their low rates of baseline apoptotic activity and the availability of sufficient protein to assess cellular signaling events. The use of neonatal cells to study molecular cardiac events is well established in the literature, and similarities between neonatal and adult cardiomyocyte response to I/R injury have been observed [1], [42]. In addition, these cells demonstrate therapeutic hypothermia protection against I/R injury and allow for further study of strategies that may mimic underlying cooling mechanisms of cardioprotection.

In summary, these results support the dual functions of p85 in modulating I/R injury. Modulating PTEN binding to 85 or PTEN phosphatase activity could have therapeutic implications in the survival of heart cells following I/R injury.
