# Correction: TAT-Protein Blockade during Ischemia/Reperfusion Reveals Critical Role for p85 PI3K-PTEN Interaction in Cardiomyocyte Injury

**DOI:** 10.1371/journal.pone.0104137

**Published:** 2014-07-25

**Authors:** The PLOS ONE Staff

There is an error in the funding statement that was introduced during the preparation of this manuscript for publication. Please refer to the correct funding statement here:

This work was supported by the Chicago Biomedical Consortium with support from the Searle Funds at the Chicago Community Trust (to T. L. Vanden Hoek and A.R. Leff), and by a grant from GlaxoSmithKline Center of Excellence (to A. R. Leff). The co-author Dr. Alan Leff has received research grant support from GlaxoSmithKline. This does not alter the authors' adherence to PLOS ONE policies on sharing data and materials. The funders had no role in study design, data collection and analysis, decision to publish, or preparation of the manuscript.
